# Analyses of key mRNAs and lncRNAs for different osteo‐differentiation potentials of periodontal ligament stem cell and gingival mesenchymal stem cell

**DOI:** 10.1111/jcmm.16571

**Published:** 2021-05-24

**Authors:** Linglu Jia, Yunpeng Zhang, Dongfang Li, Wenjing Zhang, Dongjiao Zhang, Xin Xu

**Affiliations:** ^1^ Shandong Key Laboratory of Oral Tissue Regeneration & Shandong Engineering Laboratory for Dental Materials and Oral Tissue Regeneration School and Hospital of Stomatology Cheeloo College of Medicine Shandong University Jinan China; ^2^ Department of Oral Implantology The Affiliated Stomatology Hospital of Kunming Medical University Kunming China

**Keywords:** DKK1, human gingival mesenchymal stem cells, human periodontal ligament stem cells, lncRNAs, mRNAs, osteogenic differentiation

## Abstract

Both human periodontal ligament stem cells (hPDLSCs) and human gingival mesenchymal stem cells (hGMSCs) are candidate seed cells for bone tissue engineering, but the osteo‐differentiation ability of the latter is weaker than the former, and the mechanisms are unknown. To explore the potential regulation of mRNAs and long non‐coding RNAs (lncRNAs), this study obtained the gene expression profiles of hPDLSCs and hGMSCs in both undifferentiated and osteo‐differentiated conditions by microarray assay and then analysed the common and specific differentially expressed mRNAs and lncRNAs in hPDLSCs and hGMSCs through bioinformatics method. The results showed that 275 mRNAs and 126 lncRNAs displayed similar changing patterns in hPDLSCs and hGMSCs after osteogenic induction, which may regulate the osteo‐differentiation in both types of cells. In addition, the expression of 223 mRNAs and 238 lncRNAs altered only in hPDLSCs after osteogenic induction, and 177 mRNAs and 170 lncRNAs changed only in hGMSCs. These cell‐specific differentially expressed mRNAs and lncRNAs could underlie the different osteo‐differentiation potentials of hPDLSCs and hGMSCs. Finally, dickkopf Wnt signalling pathway inhibitor 1 (DKK1) was proved to be one regulator for the weaker osteo‐differentiation ability of hGMSCs through validation experiments. We hope these results help to reveal new mRNAs‐lncRNAs‐based molecular mechanism for osteo‐differentiation of hPDLSCs and hGMSCs and provide clues on strategies for improving stem cell–mediated bone regeneration.

## 1 INTRODUCTION

The destruction of alveolar bone caused by periodontitis often leads to teeth loss, so the regeneration of bone is an essential project in the treatment of periodontitis.^1^ Recently, tissue engineering has become a new method to realize alveolar bone regeneration, which contains three elements including seed cells, grow factors and scaffold.^2^ The number and biological characteristics of seed cells directly affect the effect of bone regeneration, so many scholars pay much attention to the selection of optimal seed cells. Mesenchymal stem cells (MSCs), which own the capacities of self‐renewal and multilineage differentiation, are powerful seed cells for bone tissue engineering.^2^ Human periodontal ligament stem cells (hPDLSCs) are typical oral tissue‐derived MSCs that exist in the periodontium tissue. Since their isolation and identification in 2004,^3^ hPDLSCs have been applied in alveolar bone regeneration in different animal models.^4^, ^5^, ^6^ However, the harvest of hPDLSCs depends on tooth extraction, which limits the source of them.^7^ Human gingival mesenchymal stem cells (hGMSCs) are also oral tissue‐derived MSCs with osteogenic differentiation ability which were first reported in 2009.^8^ Compared with hPDLSCs, it is much easier to obtain enough amount of hGMSCs to meet the needs of tissue engineering, because hGMSCs could be isolated from resected gingival tissue waste in a variety of oral therapies such as gingivoplasty treatment, oral implant treatment and tooth extraction.^7^ In addition, hGMSCs were reported to own stronger proliferation ability than hPDLSCs and were not as easy as hPDLSCs to senescence after continuous passage in in vitro culture environment.^9^, ^10^, ^11^, ^12^ These characteristics make hGMSCs have great value in bone tissue engineering. However, several comparative studies on stem cells from different oral tissues by us and other scholars showed that hGMSCs are much weaker than hPDLSCs in the ability of osteogenic differentiation under same induction environment, which is not conducive to their application in bone regeneration.^9^, ^10^, ^11^, ^12^ Therefore, exploring the molecular mechanisms underlie the weaker osteo‐differentiation abilities of hGMSCs than hPDLSCs could help to find the methods of enhancing the osteogenic differentiation of hGMSCs, which will promote the application of hGMSCs in bone regeneration. However, little is known about these regulations.

Long non‐coding RNAs (lncRNAs) are RNA molecules longer than 200 nucleotides that do not encode proteins. Currently, thousands of lncRNAs have been identified in nucleus and cytoplasm of cells, and their modulator roles in gene expression and diverse biological processes are being revealed and demonstrated.^13^ Growing evidence shows that lncRNAs have influence on MSC pluripotency maintenance and multi‐differentiation.^14^, ^15^, ^16^ In oral tissue–derived MSCs, even though several lncRNAs have been reported to regulate osteo‐differentiation such as lncRNA‐POIR,^17^ lncRNA‐ANCR ^18^, ^19^ and lncRNA‐FER1L4,^20^ more new regulators need to be discovered. In addition, whether lncRNAs participate in the regulation of dissimilar osteo‐differentiation abilities among different MSCs is unknown.

With the emergence and development of microarray technologies, the large‐scale analyses of mammal transcriptome become much easier and more reliable. Comparing the molecular expressions of MSCs with different biological activities through microarray methods helps to discover potential regulatory molecules, which has been used by many scholars.^21^, ^22^, ^23^, ^24^ Our previous study provided comprehensive profiles of mRNA and lncRNA expression in hPDLSCs and hGMSCs through gene chip technology, which revealed some potential key mRNAs and lncRNAs that could regulate the different proliferation, inflammatory immunogenicity and anti‐oxidative stress abilities of hPDLSCs and hGMSCs.^25^ As to the genes that mediate the dissimilar osteo‐differentiation abilities of hPDLSCs and hGMSCs, our knowledge is still limited.

This study obtained the mRNA and lncRNA expression profiles of hPDLSCs and hGMSCs in both undifferentiated and osteo‐differentiated conditions and then focused on the same/different genes whose expression levels changed during the osteogenic differentiation in hPDLSCs and hGMSCs. We hope the results provide clues on key mRNAs and lncRNAs that regulate dissimilar osteogenic differentiation abilities in hPDLSCs and hGMSCs and then help to provide targets for improving the osteo‐differentiation ability of hGMSCs and promoting hGMSC‐based bone regeneration.

## 2 MATERIALS AND METHODS

### 2.1 Isolation and culture of hPDLSCs and hGMSCs

The experiment was approved by the Ethics Committee of Shandong University. Donors aged 16‐25 years old without systemic disease were from Stomatology Hospital of Shandong University, and informed consents were obtained from all of them for collecting fresh healthy tissue. The hPDLSCs and hGMSCs were isolated from corresponding tissues based on reported protocols.^3^, ^26^, ^27^ (a) hPDLSCs: newly extracted healthy premolars for orthodontic treatment were cleaned with phosphate‐buffered saline (PBS), and then, periodontal ligament tissues were scraped from the middle one‐third of the root surface and cut into small pieces. (b) hGMSCs: healthy gingival tissues for crown lengthening surgery were gained and cleaned with PBS, and then, the epithelial layer was removed carefully and the proper layer tissues were sliced into small patches. All above tissue pieces were digested in the solution of 3 mg/mL collagenase I (Sigma) and 4 mg/mL dispase (Sigma) at 37°C for 1 hour. Then, the suspension containing cells was seeded into culture dishes and cultured in the culture medium (a‐MEM (Corning) supplemented with 10% foetal bovine serum (Corning)) at 37°C in 5% CO_2_ incubator. The medium was refreshed every 3 days.

### 2.2 Identification of hPDLSCs and hGMSCs

Flow cytometry analyses were adopted to detect the cell surface markers of cells using BD Human MSC Analysis Kit (BD Biosciences) according to the instructions of the manufacturer. The following antibodies were used: CD90, CD44, CD105, CD73 and negative cocktail (CD34, CD11b, CD19, CD45 and HLA‐DR).

Multiple differentiation assays were used to determine the pluripotency of hPDLSCs and hGMSCs. For osteogenic induction, cells were incubated in the osteogenic medium (culture medium supplied with 10 nmol/L dexamethasone (Solarbio), 10 mmol/L β‐glycerophosphate (Solarbio), 50 mg/L ascorbic acid (Solarbio)). After 3 weeks, cells were fixed with 4% paraformaldehyde and stained with Alizarin Red, and the mineralized matrix was dissolved in 10% cetylpyridinium chloride (Solarbio). Finally, the absorbance value at 562 nm of the solution was measured using a microplate reader. For adipogenic induction, cells were cultured in adipogenic medium (culture medium supplied with 1 μmol/L dexamethasone (Solarbio), 0.2 mmol/L indomethacin (Solarbio), 0.01 g/L insulin (Solarbio), 0.5 mmol/L isobutyl‐methylxanthine(IBMX) (Solarbio)) for 3 weeks, and then, the cells were fixed with 4% paraformaldehyde and stained with Oil Red O. For chondrogenic induction, cells were centrifugated to form cell precipitation and incubated in chondrogenic medium (Cyagen) for 4 weeks, and then, the cell pellet was paraffin embedded for Alcian blue staining.

### 2.3 Alkaline phosphatase (ALP) staining and ALP activity assay

For ALP staining, the cells were fixed in 4% paraformaldehyde and then were stained using the NBT/BCIP staining kit (Beyotime) according to manufacturer's instructions. For ALP activity assay, the cells were collected for analyses using the ALP assay kit (Nanjing Jiancheng Bioengineering Institute) according to manufacturer's instructions.

### 2.4 Samples preparation and microarray analyses

Three samples of hPDLSCs, which were from three different individuals, were cultured in the osteogenic medium for 7 days as the experimental group (osteogenic induction hPDLSCs, OI hPDLSCs), and corresponding cells cultured in the culture medium for 7 days were regarded as the control group (negative control hPDLSCs, NC hPDLSCs). Similar procedures were performed for hGMSCs, and then, NC hGMSCs (n = 3) and OI hGMSCs (n = 3) were obtained. Above cells were analysed through ALP activity test and ALP staining to verify the effectiveness of osteogenesis induction. Then, all cell samples were collected and analysed through GeneChip Human Transcriptome Array 2.0 (Affymetrix) according to manufacturer's instruction by GMINIX Informatics Ltd. Co. The differentially expressed mRNAs and lncRNAs between experimental groups and control groups were filtered in compliance with significance analysis of microarray (SAM) method, and probe signals with *P* < .05 and absolute value of fold change (FC) ≥1.5 were considered to be statistically differential.^28^ FC represented the multiple of difference, and up‐regulated genes were shown as positive numbers while down‐regulated genes were expressed as negative numbers.

### 2.5 RNA extraction and quantitative real‐time PCR (qRT‐PCR)

Total RNA was extracted from cells using RNAiso Plus reagent (Takara) according to the manufacturer's instructions. Then, the RNA was reverse transcribed to cDNA by PrimeScript™ RT Reagent Kit (Takara). The qRT‐PCR was performed in a 10 μL reaction volume with TB Green (Takara) by Roche LightCycler^®^480II according to the instructions of the manufacturer. GAPDH was regarded as the internal control, and the expression of mRNAs or lncRNAs was calculated by the 2^‐△△Ct^ method. The primers of mRNAs and lncRNAs are listed in Table S1.

### 2.6 Gene Ontology (GO) analyses and pathway analyses

GO analyses were performed to identify the function of genes based on Gene Ontology Consortium database, in terms of biological process (BP), cellular component (CC) and molecular function (MF). Pathway analyses were applied to confirm what biological pathways the genes participated in based on the Kyoto Encyclopedia of Genes and Genomes (KEGG) database.

### 2.7 Construction of the coding‐non‐coding gene (CNC) co‐expression networks

The CNC networks were constructed to identify the relations between mRNAs and lncRNAs. Hybrid hierarchical clustering algorithm was used to calculate the pairwise correlation to construct a matrix for each pair, and then, the network was built based on the correlation coefficient.

### 2.8 Protein extraction and Western Blot

The cells were lysed with RIPA buffer (Solarbio) containing 1% PMSF (Solarbio) and 1% phosphatase inhibitor (Boster Bio) on ice, and then, the total protein dissolved in the supernatant was collected and denatured. The proteins were loaded to SDS‐PAGE and transferred onto PVDF membranes. The membranes were blocked with non‐fat milk and incubated with primary antibodies overnight at 4°C. Finally, the membranes were incubated with secondary antibodies and visualized using enhanced chemiluminescence reagents (Millipore). The following antibodies were used: COL1A1 (#84336), RUNX2 (ab23981, Abcam), Non‐phospho (Active) β‐Catenin (#8814), DKK1(dickkopf Wnt signalling pathway inhibitor 1) (#48367), β‐Actin (sc‐517582) and GAPDH (HRP‐60004, Proteintech). β‐Actin and GAPDH were used as internal controls. The grey value of protein bands was analysed by software ImageJ, and the fold change of target proteins was normalized by internal controls.

### 2.9 Small interfering RNA (siRNA)

The siRNA‐targeted DKK1 (siDKK1) was designed and synthesized by GenePharma to knockdown the expression of DKK1 in hGMSCs, and siRNA‐targeted none (siNC) was used as negative control. The siRNAs were transfected into cells using the Micropoly‐transfecter Cell Reagent (Micropoly) according to the manufacture's instruction. The qRT‐PCR or Western blot was used to detect the knockdown efficiency of DKK1. In the following experiments, siNC hGMSCs and siDKK1 hGMSCs were cultured in the osteogenic medium for 7 days after transfection. During these 7 days, siNC hGMSCs and siDKK1 hGMSCs were transfected with corresponding siRNAs again on the 4th day to ensure the continuous knockdown of DKK1.

The sequences of siRNAs are listed below: siNC: Sense 5′‐UUCUCCGAACGUGUCACGUTT‐3′, Antisense 5′‐ACUUGACACGUUCGGAGAATT‐3′. siDKK1: Sense 5′‐GCCGGAUACAGAAAGAUCATT‐3′, Antisense 5′‐UGAUCUUUCUGUAUCCGGCTT‐3′.

### 2.10 Statistical analyses

All assays were repeated at least three times, and data were presented as mean ± standard deviations. Statistical analyses were carried out using SPSS 19.0, and independent‐sample *t* tests, Fisher exact test and Pearson correlation were used to determine statistical differences. The *P* < .05 was considered statistically significant.

## 3 RESULTS

### 3.1 Culture and identification of hPDLSCs and hGMSCs

**FIGURE 1:**
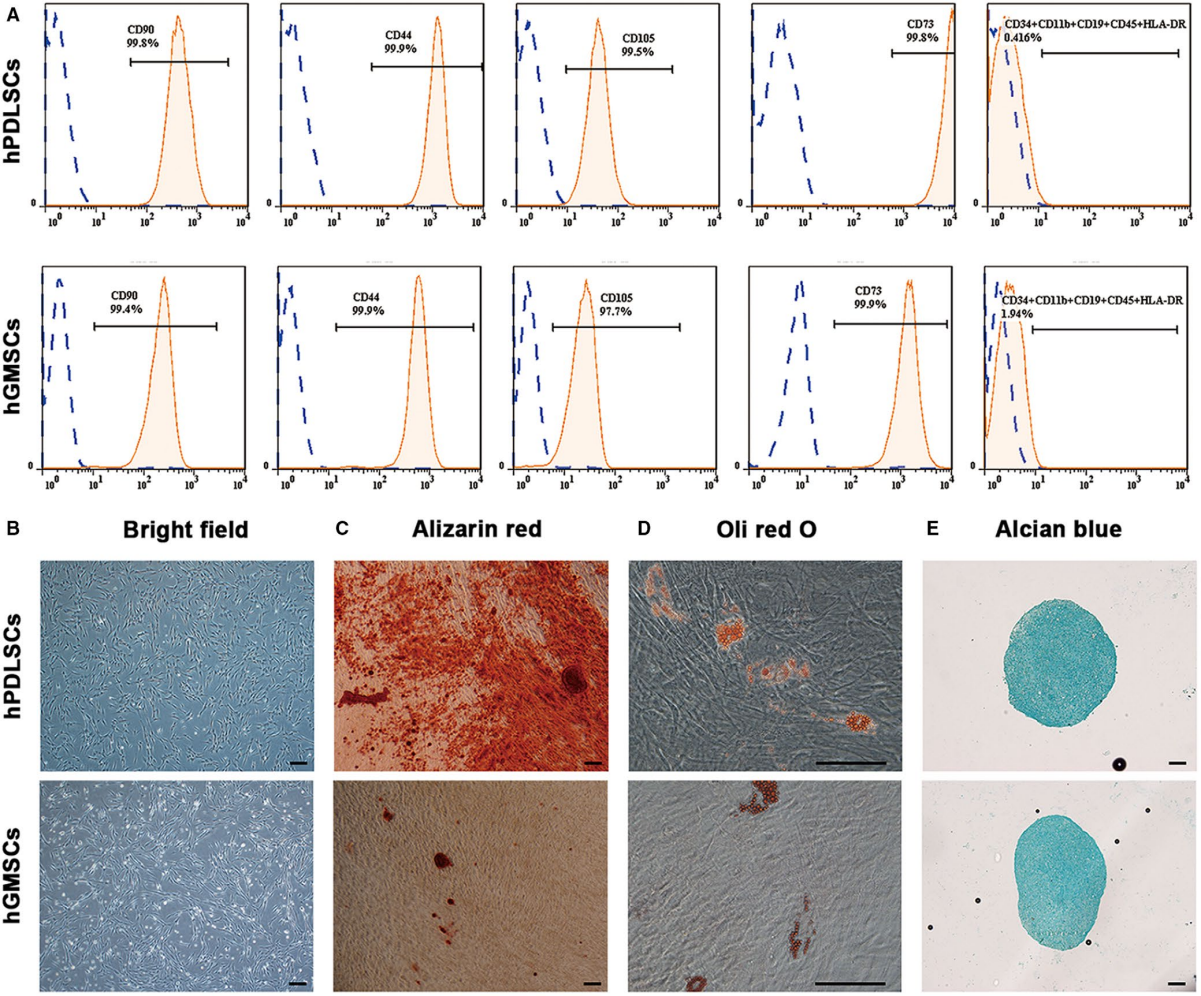
Culture and identification of hPDLSCs and hGMSCs (A) Flow cytometry analyses of MSC‐specific surface markers (CD90, CD44, CD105 and CD73) and endothelial cell‐specific markers (CD34, CD11b, CD19, CD45 and HLA‐DR) (n = 3) (B) The cell morphology of hPDLSCs and hGMSCs when cultured in vitro. (C) Alizarin Red staining after osteogenic induction for 3 wk. (n = 3) (D) Oil Red O staining after adipogenic induction for 3 wk. (n = 3) (E) Alcian Blue staining after chondrogenic induction for 4 wk. (n = 3) Scale bar = 200 μm

Both hPDLSCs and hGMSCs presented spindle‐shaped morphology (Figure 1B). They both were positive to MSC‐specific surface markers (CD90, CD44, CD105 and CD73), but negative to hematopoietic and endothelial cell–specific markers (CD34, CD11b, CD19, CD45 and HLA‐DR) (Figure 1A). The result of Alizarin Red (Figure 1C), Oil Red O (Figure 1D) and Alcian Blue staining (Figure 1E) indicated that both hPDLSCs and hGMSCs could differentiate into osteoblasts, adipocytes and chondrocytes.

### 3.2 Quality control of gene chip microarray assay

**FIGURE 2:**
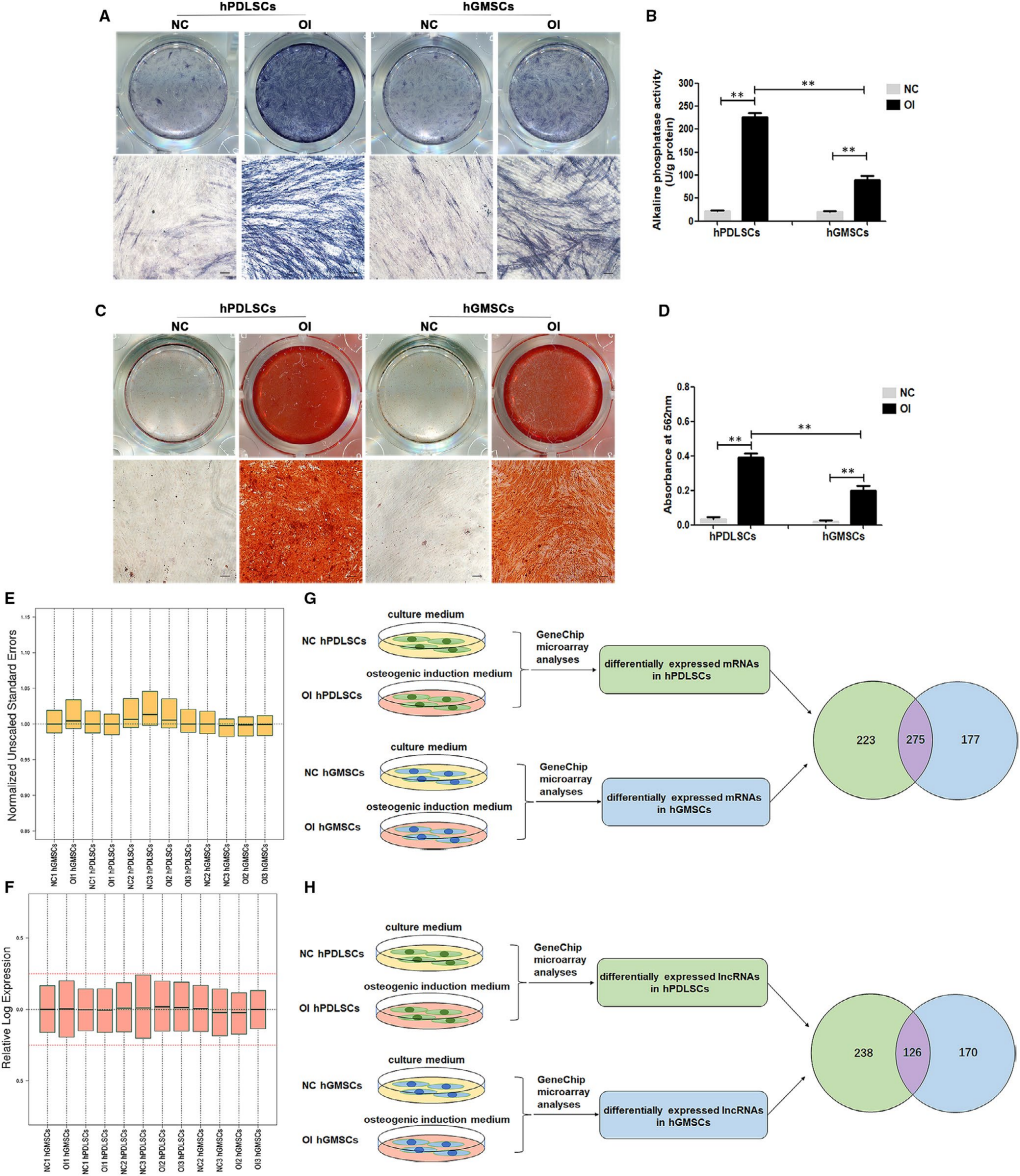
hPDLSCs and hGMSCs showed different osteogenic differentiation potential and were analysed by microarray assay (A) ALP staining of hPDLSCs and hGMSCs that were cultured in culture medium (NC) and osteogenic medium (OI) for 7 d. (n = 3) Scale bar = 200 μm. (B) ALP activity analyses of hPDLSCs and hGMSCs that were cultured in culture medium (NC) and osteogenic medium (OI) for 7 d. (n = 3) (C) Alizarin Red staining of hPDLSCs and hGMSCs that were cultured in culture medium (NC) and osteogenic medium (OI) for 21 d. (n = 3) Scale = 200 μm. (D) Quantitative analysis of mineralized nodules in hPDLSCs and hGMSCs that were cultured in culture medium (NC) and osteogenic medium (OI) for 21 d. (n = 3) (E) Quality evaluation of microarray assay by NUSE (normalized unscaled standard errors) method. (F) Quality evaluation of microarray assay by RLE (relative log expression) method. (G) The common and specific differentially expressed mRNAs in hPDLSCs and hGMSCs after osteogenic induction were filtered. (H) The common and specific differentially expressed lncRNAs in hPDLSCs and hGMSCs after osteogenic induction were filtered. **P < .01

After cultured in osteogenic medium for 7 days, the ALP activity of OI hPDLSCs is much higher than NC hPDLSCs (Figure 2B), and the ALP staining of OI hPDLSCs is much deeper than NC hPDLSCs (Figure 2A). Similarly, OI hGMSCs had higher ALP activity (Figure 2B) and deeper ALP staining (Figure 2A) than NC hGMSCs. These results indicated that the osteogenic induction is effective, and these cell samples could be sent for gene chip microarray assay.

Notably, when comparing OI hPDLSCs and OI hGMSCs, the ALP activity and ALP staining is stronger in the former than the latter (Figure 2A,B), although they were under the same induction conditions. We also compared the formation of mineralized matrix in OI hPDLSCs and OI hGMSCs after osteogenic induction for 21d. As shown in Figure 2C, the Alizarin Red staining is stronger in OI hPDLSCs than in OI hGMSCs. The quantitative analysis of mineralized matrix also showed that there was more mineralized matrix in OI hPDLSCs than in OI hGMSCs (Figure 2D). These results supported that hPDLSCs had stronger osteogenic differentiation ability than hGMSCs, which was consistent with previous studies.^9^, ^10^, ^11^, ^12^, ^29^

In gene chip microarray assay, the assay quality was controlled based on NUSE (normalized unscaled standard errors) and RLE (relative log expression) method. As shown in Figure 2E,F, the median of NUSE meets (1‐0.2) < median (NUSE) < (1 + 0.2), and the median of RLE meets −0.25 < median (RLE) < 0.25, suggesting all cell samples and microarray assay were qualified.

### 3.3 Comparison of mRNA expression profiles between hPDLSCs and hGMSCs after osteogenic induction

Using the method of microarray assay, we obtained the mRNA expression profiles of NC hPDLSCs, OI hPDLSCs, NC hGMSCs and OI hGMSCs. After separately comparing NC hPDLSCs and OI hPDLSCs, NC hGMSCs and OI hGMSCs, we got the mRNAs whose expression levels were altered after osteogenic induction in hPDLSCs and hGMSCs. Then, we compared above differentially expressed mRNAs of hPDLSCs and hGMSCs to screen potential important regulators (Figure 2G).

Firstly, the expression of 275 mRNAs showed similar changes in both hPDLSCs and hGMSCs after osteogenic induction, including 123 up‐regulated mRNAs and 152 down‐regulated mRNAs (Figure 2G, Table S2). We speculated that these common differentially mRNAs were likely to play regulatory roles in the osteogenic differentiation of both hPDLSCs and hGMSCs. According to the absolute value of FC, the top 10 common differentially expressed mRNAs after osteogenic induction in hPDLSCs and hGMSCs are listed in Table 1. Next, we screened specific differentially expressed mRNAs in hPDLSCs or in hGMSCs. The results showed that the expression of 223 mRNAs altered only in hPDLSCs after osteogenic induction, among which 93 were up‐regulated and 130 were down‐regulated (Figure 2G, Table S3). While the expression of 177 mRNAs altered only in hGMSCs after osteogenic induction, including 98 up‐regulated ones and 79 down‐regulated ones (Figure 2G, Table S4). We speculated that these specific differentially expressed mRNAs were related to the different osteo‐differentiation abilities of hPDLSCs and hGMSCs, which need further experiments to validate. According to the absolute value of FC, the top 10 specific differentially expressed mRNAs after osteogenic induction in hPDLSCs and hGMSCs are listed in Table 1.

**TABLE 1:** Top 10 common and specific differentially expressed mRNAs in hPDLSCs and hGMSCs after osteogenic induction

Features	Gene symbol	Accession number	Database source	Fold change in hPDLSCs	*P*‐value in hPDLSCs	Fold change in hGMSCs	*P*‐value in hGMSCs
Common differentially expressed mRNAs in hPDLSCs and hGMSCs	PIP	NM_002652	RefSeq	23.35823	.000049	16.87401	.002367
	PSAT1	NM_058179	RefSeq	−20.49963	.000049	−6.372215	.003360
	FKBP5	NM_004117	RefSeq	16.61331	.000049	12.18578	.000074
	CORIN	NM_006587	RefSeq	8.569042	.001502	8.805004	.000356
	SLC7A5	NM_003486	RefSeq	−8.110064	.000049	−3.876736	.011785
	ASNS	NM_001178075	RefSeq	−6.414056	.000098	−3.673525	.005114
	APOD	NM_001647	RefSeq	6.131639	.000073	4.241052	.000074
	FRZB	NM_001463	RefSeq	5.955356	.000049	3.290549	.000061
	VLDLR	NM_001018056	RefSeq	−5.514227	.000049	−3.73504	.000650
	SEMA3D	NM_152754	RefSeq	−5.212781	.000098	−3.888794	.002588
Specific differentially expressed mRNAs in hPDLSCs	VCAM1	NM_001078	RefSeq	−7.947481	.000452	‐	‐
	ADH1B	NM_000668	RefSeq	7.399272	.005998	‐	‐
	TNFSF18	NM_005092	RefSeq	−4.718332	.016857	‐	‐
	STC1	NM_003155	RefSeq	4.414963	.001136	‐	‐
	ADAMTS3	NM_014243	RefSeq	−3.375889	.009675	‐	‐
	KRTAP1‐5	NM_031957	RefSeq	−2.82819	.010371	‐	‐
	CLDN1	NM_021101	RefSeq	−2.628763	.004117	‐	‐
	SLIT2	NM_004787	RefSeq	−2.451802	.000147	‐	‐
	KIT	NM_000222	RefSeq	−2.404533	.00011	‐	‐
	RGS4	NM_001102445	RefSeq	2.312218	.035791	‐	‐
Specific differentially expressed mRNAs in hGMSCs	FNDC1	NM_032532	RefSeq	‐	‐	−6.731615	.016727
	SLC7A2	NM_001008539	RefSeq	‐	‐	5.026267	.037146
	DKK1	NM_012242	RefSeq	‐	‐	4.990853	.000907
	CYP7B1	NM_004820	RefSeq	‐	‐	3.678479	.028623
	FRAS1	NM_001166133	RefSeq	‐	‐	2.670758	.000895
	TRPC6	NM_004621	RefSeq	‐	‐	2.618061	.014324
	HMGCS1	NM_001098272	RefSeq	‐	‐	2.572768	.034791
	BIRC3	NM_001165	RefSeq	‐	‐	2.536337	.032669
	CXCL14	NM_004887	RefSeq	‐	‐	−2.441522	.000074
	BMP4	NM_001202	RefSeq	‐	‐	−2.39276	.031934

### 3.4 Comparison of lncRNAs expression profiles between hPDLSCs and hGMSCs after osteogenic induction

Using the method similar to mRNA analyses, we compared and analysed the lncRNAs expression profile of NC hPDLSCs, OI hPDLSCs, NC hGMSCs and OI hGMSCs and screened common and specific differentially expressed lncRNAs in hPDLSCs and hGMSCs after osteogenic induction (Figure 2H).

Among these lncRNAs, the expression of 126 lncRNAs changed in similar ways in hPDLSCs and hGMSCs; of those, 66 were up‐regulated and 60 were down‐regulated (Figure 2H, Table S5). The top 10 of these common differentially expressed lncRNAs after osteogenic induction in hPDLSCs and hGMSCs are listed in Table 2. We speculated that these common differentially expressed lncRNAs possibly participated in the regulation of osteo‐differentiation in both hPDLSCs and hGMSCs. At the same time, the expression of some lncRNAs changed only in one type of cell after osteogenic induction, including 238 lncRNAs in hPDLSCs and 170 lncRNAs in hGMSCs (Figure 2H): in hPDLSCs, 134 out of 238 specific differentially expressed lncRNAs were up‐regulated and 104 were down‐regulated (Table S6); while in hGMSCs, 109 out of 170 specific differentially expressed lncRNAs were up‐regulated and 61 were down‐regulated (Table S7). According to the absolute value of FC, the top 10 specific differentially expressed lncRNAs after osteogenic induction in hPDLSCs and hGMSCs are listed in Table 2. These specific differentially expressed lncRNAs in hPDLSCs and hGMSCs were speculated to be related with the dissimilar osteogenic differentiation ability of hPDLSCs and hGMSCs and required further study.

**TABLE 2:** Top 10 common and specific differentially expressed lncRNAs in hPDLSCs and hGMSCs after osteogenic induction

Features	Gene symbol	Accession number	Database source	Fold change in hPDLSCs	*P*‐value in hDPSCs	Fold change in hGMSCs	*P*‐value in hGMSCs
Common differentially expressed lncRNAs in hPDLSCs and hGMSCs	PSAT1P4	ENST00000454471	ENSEMBL	−7.124656	.000025	−4.782379	.000139
	‐	n341111	NONCODE	−6.068412	.000155	−4.653776	.003069
	‐	n385115	NONCODE	−5.525836	.003576	−3.632913	.001139
	‐	TCONS_00016019‐XLOC_007414	Rinn lincRNA	5.209193	.000056	6.274862	.000025
	‐	n407850	NONCODE	−5.199306	.000025	−2.510003	.003943
	‐	n339452	NONCODE	−4.417426	.000025	−3.595392	.004683
	‐	n410329	NONCODE	−4.108275	.000025	−1.988969	.002848
	‐	n342706	NONCODE	4.026074	.00854	3.191165	.016985
	‐	n334375	NONCODE	−3.916899	.000218	−2.642809	.004879
	MT1L	NR_001447	RefSeq	3.362156	.000056	2.705894	.013916
Specific differentially expressed lncRNAs in hPDLSCs	‐	n340869	NONCODE	−4.171751	.008969	‐	‐
	‐	n333720	NONCODE	−3.771858	.013410	‐	‐
	‐	n334500	NONCODE	−2.898203	.000124	‐	‐
	‐	ENST00000401164	ENSEMBL	2.717534	.000050	‐	‐
	‐	n338232	NONCODE	−2.711309	.044527	‐	‐
	‐	ENST00000408172	ENSEMBL	2.657157	.000746	‐	‐
	‐	n334116	NONCODE	−2.650476	.034700	‐	‐
	‐	ENST00000408883	ENSEMBL	2.593688	.000211	‐	‐
	‐	TCONS_00024775‐XLOC_012050	Rinn lincRNA	2.570061	.001375	‐	‐
	‐	n339176	NONCODE	‐	‐	‐	‐
Specific differentially expressed lncRNAs in hGMSCs	‐	n334786	NONCODE	‐	‐	−3.712912	.009771
	‐	n339913	NONCODE	‐	‐	−3.323282	.000278
	‐	n334700	NONCODE	‐	‐	3.284611	.005335
	‐	n341766	NONCODE	‐	‐	−2.712707	.000209
	‐	n338032	NONCODE	‐	‐	−2.461361	.016093
	‐	n334561	NONCODE	‐	‐	2.413538	.000228
	‐	n334095	NONCODE	‐	‐	2.278296	.022288
	‐	ENST00000549251	ENSEMBL	‐	‐	2.228193	.018295
	‐	n338015	NONCODE	‐	‐	−2.162764	.012688
	‐	n335743	NONCODE			2.216221	.006803

### 3.5 Validation of lncRNAs and mRNAs expression by qRT‐PCR

Based on the FC, *P*‐value and nucleotide sequence, 9 mRNAs were selected for validation in hPDLSCs and hGMSCs, including 3 common differentially expressed mRNAs and 3 specific differentially expressed mRNAs in each type of cell. Similarly, 9 lncRNAs were selected for validation in hPDLSCs and hGMSCs, including 3 common differentially expressed lncRNAs and 3 specific differentially expressed lncRNAs in each type of cell.

**FIGURE 3:**
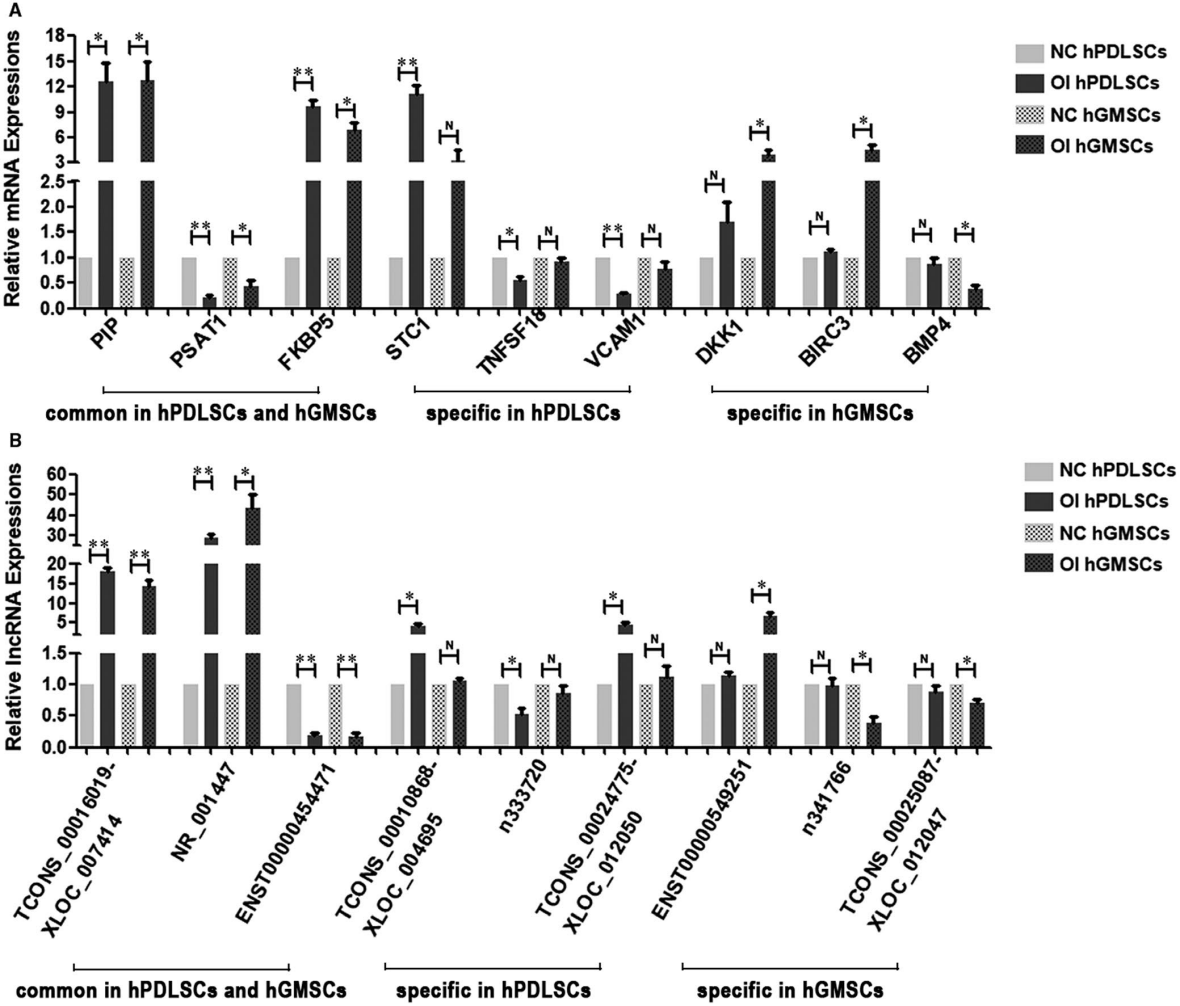
Validation of the expression of mRNAs and lncRNAs in hPDLSCs and hGMSCs by qRT‐PCR (A) qRT‐PCR was used to validate the expression data of mRNAs from microarray analyses, including the common differentially expressed ones (PIP, PSAT1, FKBP5), the specific differentially expressed ones in hPDLSCs (STC1, TNFSF18, VCAM1), and the specific differentially expressed ones in hGMSCs (DKK1, BIRC3 and BMP4). (n = 3) (B) qRT‐PCR was used to validate the expression data of lncRNAs from microarray analyses, including the common differentially expressed ones (TCONS_00016019‐XLOC_007414, NR_001447, ENST00000454471), the specific differentially expressed ones in hPDLSCs (TCONS_00010868‐XLOC_004695, n333720, TCONS_00024775‐XLOC_012050) and the specific differentially expressed ones in hGMSCs (ENST00000549251, n341766 and TCONS_00025087‐XLOC_012047). (n = 3) *P < .05, **P < .01, N *P* > .05

The qRT‐PCR was used to detect the expression of above selected mRNAs and lncRNAs in hPDLSCs and hGMSCs that were cultured in osteogenic medium and in culture medium for 7 days. The results showed that qRT‐PCR and microarray analyses were consistent in hPDLSCs and hGMSCs, which suggested that the data of microarray analyses were credible (Figure 3).

### 3.6 Function analyses of common differentially expressed mRNAs in hPDLSCs and hGMSCs

GO analyses and pathway analyses were carried out to analyse the function of 275 common differentially expressed mRNAs in hPDLSCs and hGMSCs.

**FIGURE 4:**
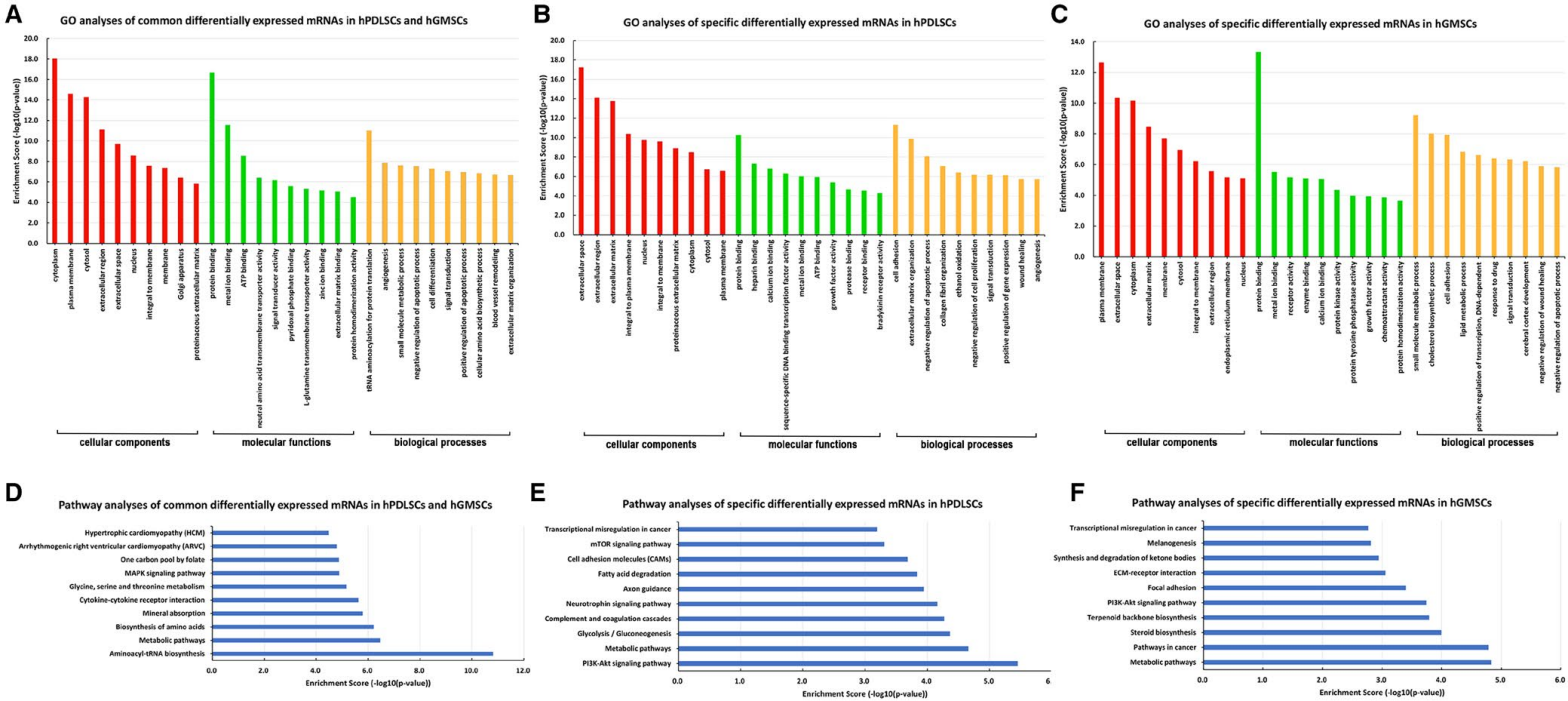
GO and pathway analyses of differentially expressed mRNAs in hPDLSCs and hGMSCs (A‐C) GO analyses of the common differentially expressed mRNAs (A), specific differentially expressed mRNAs in hPDLSCs (B), and specific differentially expressed mRNAs in hGMSCs (C) after osteogenic induction. The x‐axis represents the GO categories (biological process (BP), cellular component (CC) and molecular function (MF)) and the y‐axis represents the enrichment score (‐log 10 (*P*‐value)). (D‐F) Pathway analyses of the common differentially expressed mRNAs (D), specific differentially expressed mRNAs in hPDLSCs (E) and specific differentially expressed mRNAs in hGMSCs (F) after osteogenic induction. The x‐axis shows the enrichment score (‐log 10 (*P*‐value)) and the y‐axis shows the pathway categories

In GO analyses, these common differentially expressed mRNAs were mainly enriched in 187 BP terms, 47 MF terms and 48 CC terms (Table S8). BP enrichment was found in tRNA aminoacylation for protein translation, small molecule metabolic process, cell differentiation and so on. MF enrichment mainly distributed in terms like protein binding, ATP binding, signal transducer activity and so on. CC enrichment focused in terms including cytoplasm, extracellular region and extracellular space. The top 10 GO terms (BP, CC, MF) are shown in Figure 4A according to enrichment score (‐log _10_ (*P*‐value)).

Pathway analyses showed that these mRNAs were significantly enriched in 57 signalling pathways including aminoacyl‐tRNA biosynthesis pathway, metabolic pathways, biosynthesis of amino acid pathway, MAPK signalling pathway and so on (Figure 4D, Table S9). Notably, MAPK signalling pathway has been reported to be a classical pathway that regulates the osteogenic differentiation of MSCs,^30^, ^31^ so we speculated that this pathway could play important roles in hPDLSCs and hGMSCs during osteogenic differentiation. The top 10 pathways are shown in Figure 4D according to enrichment score.

### 3.7 Function analyses of specific differentially expressed mRNAs in hPDLSCs and hGMSCs

GO analyses and pathway analyses were carried out to analyse the function of 223 specific differentially expressed mRNAs in hPDLSCs and 177 specific differentially expressed mRNAs in hGMSCs.

In hPDLSCs, 223 specific differentially expressed mRNAs after osteogenic induction were significantly enriched for 106 BP terms, 38 MF terms and 21 CC terms (Table S10). Among them, BP such as cell adhesion, negative regulation of apoptotic process and extracellular matrix organization had the most significant difference; MF such as protein binding, heparin binding and calcium ion binding had the most significant difference; CC such as extracellular space, extracellular matrix and extracellular region had the most significant difference. These results suggested that a large proportion of these differentially expressed genes located in extracellular space and participated in extracellular matrix organization, which were closely related to osteogenic differentiation and biomineralization of cells. In addition, these mRNAs were involved in 51 signalling pathways, such as PI3K‐Akt signalling pathway, metabolic pathways, glycolysis/gluconeogenesis pathways and mTOR signalling pathway (Table S11). Among these pathways, PI3K‐Akt signalling pathway, mTOR signalling pathway and so on have been reported to be positive regulators of osteogenic differentiation in MSCs,^30^, ^31^ so we speculated that the stronger osteo‐differentiation ability of hPDLSCs could be regulated by these signalling pathways. The top 10 GO terms (BP, CC, MF) and pathway terms are shown in Figure 4B and Figure 4E according to enrichment score.

In hGMSCs, 177 specific differentially expressed mRNAs after osteogenic induction were significantly enriched for 253 BP terms, 64 MF terms and 41 CC terms (Table S12). For example, these mRNAs were enriched in CC terms such as extracellular space and extracellular matrix, which may affect the extracellular matrix mineralization. Pathway analyses suggested that these mRNAs distributed in 53 signalling pathways including metabolic pathways, steroid biosynthesis pathway, terpenoid backbone biosynthesis pathway and so on (Table S13). It was worth noting that some osteo‐differentiation‐related pathways such as PI3K‐Akt signalling pathway and Wnt signalling pathway had quite high enrichment score (Table S13), so we speculated that they may be related to the weaker osteo‐differentiation ability of hGMSCs. The top 10 GO terms (BP, CC, MF) and pathway terms are shown in Figure 4C and Figure 4F according to enrichment score.

### 3.8 Function prediction of differentially expressed lncRNAs via CNC networks

**FIGURE 5:**
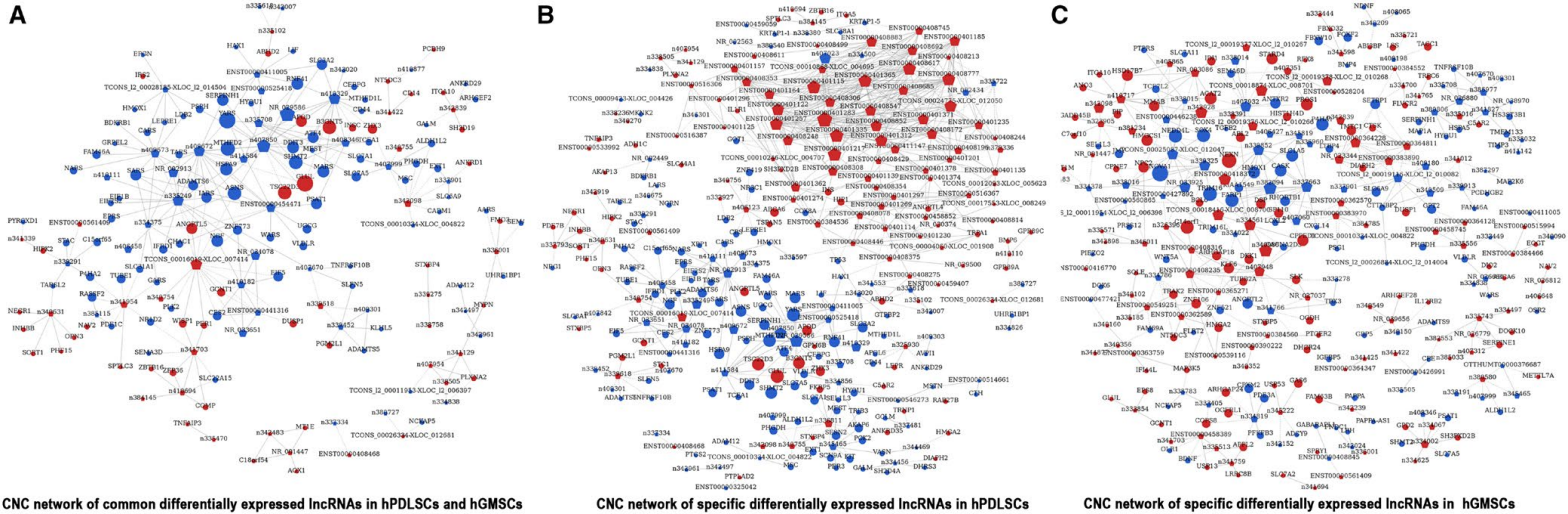
CNC networks of differentially expressed mRNAs and lncRNAs in hPDLSCs and hGMSCs. The common differentially expressed lncRNAs(A), the specific differentially expressed lncRNAs in hPDLSCs(B) and the specific differentially expressed mRNAs in hGMSCs (C) after osteogenic induction was used to build CNC networks. Circular nodes represent mRNAs and pentagonal nodes represent lncRNAs (Red: up‐regulated genes. Blue: down‐regulated genes). The size of nodes represents number of connectivity, with larger sizes indicating the nodes have connection with more nodes. Positive correlations are shown with solid lines, while dashed lines represent negative correlations

As mentioned above, the expression of 126 lncRNAs changed in similar ways in hPDLSCs and hGMSCs after osteogenic induction, and the expression of 238 lncRNAs in hPDLSCs and 170 lncRNAs in hGMSCs altered only in one type of cells. Based on CNC analyses, these common differentially expressed lncRNAs constructed 297 co‐expression relationships with differentially expressed mRNAs (Figure 5A), while the specific differentially expressed lncRNAs in hPDLSCs and hGMSCs constructed 637 (Figure 5B) and 415 (Figure 5C) co‐expression relationships separately. These co‐expression relationships provided clues for the prediction of function and molecular mechanism of lncRNAs in hPDLSCs and hGMSCs.

### 3.9 DKK1 underlies the weaker osteogenic differentiation ability of hGMSCs as compared to hPDLSCs

According to the results of microarray assay and bioinformatics analysis, gene DKK1, which belongs to specific differentially expressed mRNAs in hGMSCs, attracted our attention. The protein encoded by gene DKK1 is dickkopf Wnt signalling pathway inhibitor 1 (DKK1), which binds to the receptor and inhibits β‐catenin‐dependent Wnt signalling.^32^, ^33^ Since Wnt signalling pathway has been reported to be a positive regulator of the osteogenic differentiation, we speculated that the up‐regulation of DKK1 in hGMSCs was related with the weaker osteo‐differentiation ability of hGMSCs. To validate this speculation, we carried out the following experiments.

**FIGURE 6:**
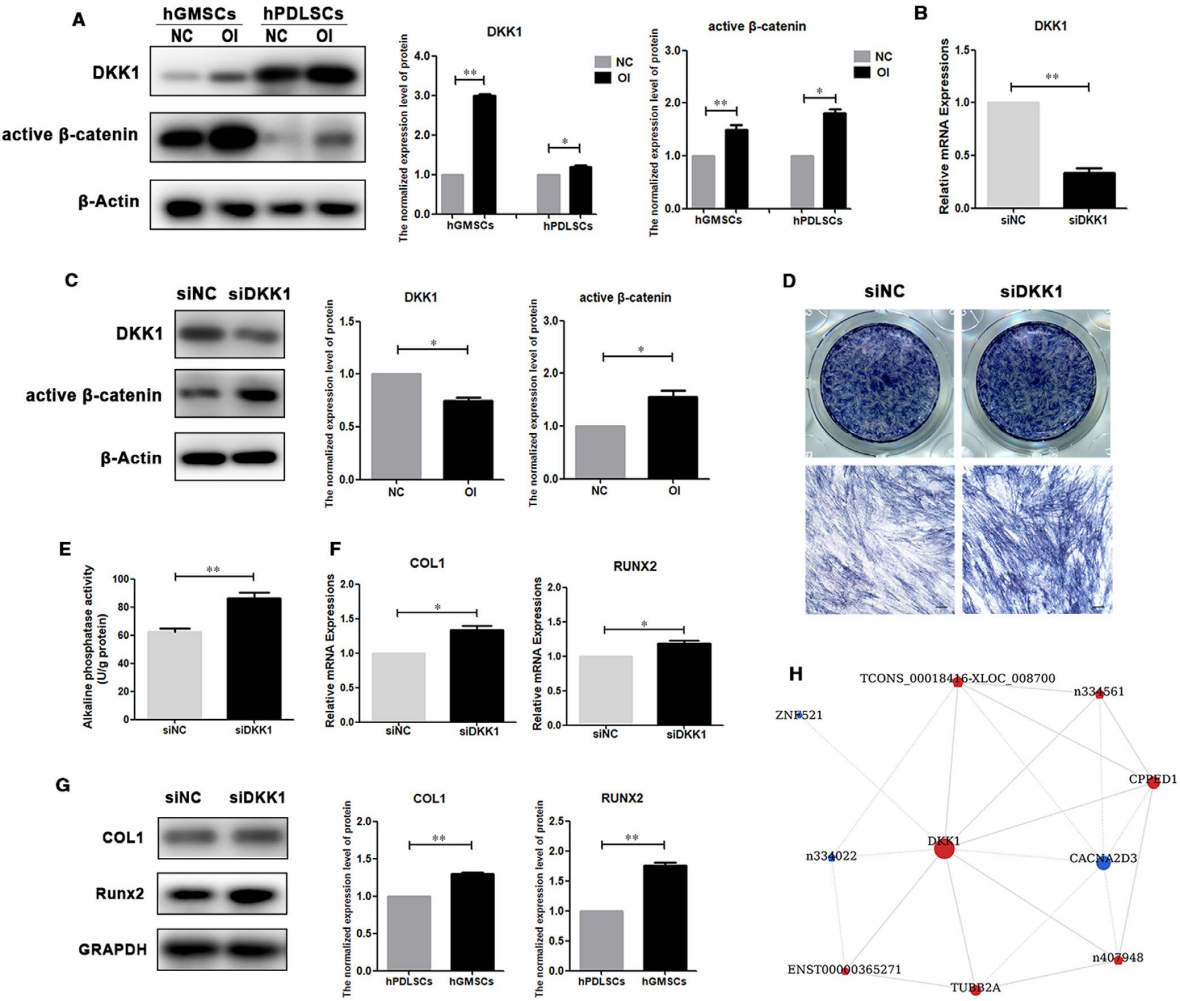
DKK1 is one of the regulators that mediated dissimilar osteogenic differentiation abilities of hPDLSCs and hGMSCs (A) The protein level of DKK1 and active β‐catenin in hPDLSCs and hGMSCs that were cultured in culture medium (NC) and osteogenic medium (OI) for 7 d was detected by Western Blot. The histograms represent the relative expression levels of proteins normalized to β‐Actin. (n = 3) (B) The mRNA level of DKK1 in hGMSCs that were transfected with siDKK1 for 48 h was detected by qRT‐PCR. (n = 3) (C) The protein level of DKK1 in hGMSCs that were transfected with siDKK1 for 72 h was detected by Western blot. The histograms represent the relative expression levels of proteins normalized to β‐Actin. (n = 3) (D) ALP staining of siNC hGMSCs and siDKK1 hGMSCs after osteogenic induction for 7 d. Scale bar = 200 μm. (n = 3) (E) ALP activity assay of siNC hGMSCs and siDKK1 hGMSCs after osteogenic induction for 7 d. (n = 3) (F) The mRNA level of COL1 and Runx2 in siNC hGMSCs and siDKK1 hGMSCs after osteogenic induction for 7 d. (n = 3) (G) The protein level of COL1 and Runx2 in siNC hGMSCs and siDKK1 hGMSCs after osteogenic induction for 7 d. The histograms represent the relative expression levels of proteins normalized to GAPDH. (n = 3) (H) The CNC network with DKK1 as the centre in hGMSCs. *P < .05, **P < .01

Firstly, we detected the expression of DKK1 in hPDLSCs and hGMSCs after osteogenic induction for 7 days. The results showed that the protein level of DKK1 in hGMSCs was significantly up‐regulated after osteogenic induction. As for hPDLSCs, even though the protein level of DKK1 was also up‐regulated after osteogenic induction, the degree of this up‐regulation is much smaller than that of hGMSCs (Figure 6A). Since DKK1 was reported as an inhibitor of classical Wnt signalling pathway, we also detected the expression of active β‐catenin, a key molecule of this pathway. The results showed that the protein of active β‐catenin was increased in both hPDLSCs and hGMSCs after osteogenic induction; however, the increase of active β‐catenin in hGMSCs is less than that in hPDLSCs, suggesting the activation of classical Wnt signalling pathway in hGMSCs after osteogenic induction was less than that in hPDLSCs (Figure 6A).

Next, we used siRNA to reduce the expression of DKK1 in hGMSCs. The results of qRT‐PCR (Figure 6B) and Western blot (Figure 6C) showed that the interference of DKK1 was successful. Then, siNC hGMSCs and siDKK1 hGMSCs were osteogenic inducted for 7 days. As shown in Figure 6, when DKK1 was interfered, the ALP activity increased (Figure 6E), the ALP staining became darker (Figure 6D), and the mRNA and protein level of osteogenic differentiation markers such as COL1 and Runx2 increased (Figure 6F,G). These results proved that interfering DKK1 promoted the osteogenic differentiation of hGMSCs. Thus, it can be seen that DKK1 could be one of the genes that underlies the weaker osteogenic differentiation ability of hGMSCs as compared to hPDLSCs.

Finally, we set up a CNC network with DKK1 as the centre in hGMSCs to screen lncRNAs that related to DKK1. The results showed that the expression of lncRNA ENST00000365271, n407948, TCONS_00018416‐XLOC_008700 and n334561 were positively correlated with DKK1, and the expression of lncRNA n334022 was negatively correlated with DKK1. Whether these lncRNAs cooperated with DKK1 to affect the osteogenic differentiation of hGMSCs required further study.

## 4 DISCUSSION

Various studies showed that hPDLSCs and hGMSCs display different osteo‐differentiation potential under the same induction condition,^9^, ^10^, ^11^, ^12^ which could be caused by some intracellular regulations mediated by proteins and non‐coding mRNAs. To explore the underlying mechanisms, the present study compared the alteration patterns of gene expression profiles between hPDLSCs and hGMSCs after osteogenic induction through microarray method. As expected, some common and specific differentially expressed mRNAs and lncRNAs in hPDLSCs and hGMSCs were screened out, proving that there are different mRNA‐lncRNA‐based regulations in the two types of cells.

The expressions of several mRNAs and lncRNAs had the same trend of change in hPDLSCs and hGMSCs after osteogenic induction, suggesting these mRNAs and lncRNAs could function in the process of osteogenic differentiation in both hPDLSCs and hGMSCs. Among these mRNAs, PIP, FKBP5, PSAT1 and so on have larger fold change. Prolactin‐induced protein (PIP) is a 17‐kDa single polypeptide chain secreted by many normal apocrine cells. Previous studies proved that PIP was rich in breast tumours and could regulate adhesion,^34^ proliferation^35^ and invasion^36^ of tumour cells. FK506‐binding protein 5 (FKBP5) was reported to participate in the regulation of steroid hormone receptor, stress‐related mental disease, cancer and other physiological or pathological processes.^37^ Phosphoserine aminotransferase 1 (PSAT1) has been proved to involve in the development of a variety of tumours^38^, ^39^ and affect the fate of mouse embryonic stem cells.^40^ Up to now, few study has explored the relationship between above differentially expressed genes and osteogenesis of MSCs, so whether they have effects on the differentiation potency of hPDLSCs and hGMSCs need further study. Pathway analyses and GO analyses revealed that these common differentially expressed mRNAs were enriched in metabolic pathways, biosynthesis of amino acids, MAPK signalling pathway and so on, suggesting these pathways and biological process could be quite important for the osteo‐differentiation of hPDLSCs and hGMSCs. Notably, MAPK signalling pathway is reported to be a classical signal axis that could regulate the osteogenic differentiation of MSCs,^41^, ^42^ which supports our results. As for the common differentially expressed lncRNAs in hPDLSCs and hGMSCs, some of them have been studied to some extent, such as NR_038849 were reported to sponge miR‐422a to aggravate the tumorigenesis of human osteosarcoma.^43^ However, the knowledge about most of these lncRNAs seems to limit to their location and sequence, and whether they play roles in MSCs needs further investigation.

To explore the molecular mechanisms of dissimilar osteo‐differentiation potential of hPDLSCs and hGMSCs, we focused on the specific differentially expressed mRNAs and lncRNAs in hPDLSCs or hGMSCs after osteogenic induction. Among these genes, DKK1, the expression of which increased more in hGMSCs than in hPDLSCs after osteogenic induction, is an inhibitor of classical Wnt signalling pathway.^32^, ^33^ In this pathway, a series of signal transduction finally promotes non‐phospho β‐catenin (active β‐catenin) to transport into nucleus to function.^32^ Various studies have shown that Wnt signalling pathway is a positive regulator of MSCs osteo‐differentiation.^44^, ^45^, ^46^ Therefore, we speculated that the increased expression of DKK1 was one of the reasons for the weaker osteo‐differentiation potential of hGMSCs as compared to hPDLSCs. The following experiments showed that interfering the expression of DKK1 could effectively promote the osteogenic differentiation of hGMSCs, which verified our speculation. In fact, some previous studies showed that anti‐DKK1 could enhance the osteogenic differentiation of stem cells,^47^, ^48^ which were consistent with our finding. As to why the expression of DKK1 was more significantly up‐regulated in hGMSCs than in hPDLSCs after osteogenic induction, some intracellular regulations such as epigenetic modification and signal transduction networks could be potential reasons, which will be our future research directions. We also screened lncRNAs that had co‐expression relationships with DKK1 by CNC network, which provided clues for seeking for lncRNAs that underlies the weaker osteo‐differentiation ability of hGMSCs. Anyway, inhibiting the expression of DKK1 could be an effective method to improve the oste‐differentiation ability of hGMSCs.

In addition to DKK1, other specific differentially expressed mRNAs in hPDLSCs or hGMSCs after osteogenic induction may also affect the different osteo‐differentiation potential of two types of cells, which requires further validation. For example, the mRNA of BMP4, which was reported to be a positive regulator of MSCs osteogenic differentiation,^49^, ^50^ significantly decreased only in hGMSCs after osteogenic induction. SCT1, a gene was reported to promote the osteogenic differentiation of osteoblast,^51^, ^52^ was up‐regulated only in hPDLSCs after osteogenic induction. These mRNAs are potential future research targets. In addition, some lncRNAs were specifically differentially expressed in hPDLSCs or hGMSCs after osteogenic induction. It was reasonable to infer that these lncRNAs play roles in the process of osteogenic differentiation in hPDLSCs or hGMSCs, especially in the different osteo‐differentiation potential of the two types of cells. Anyway, the result of the present study was a preliminary investigation for the molecular mechanisms of dissimilar osteo‐differentiation capabilities of hPDLSCs and hGMSCs, and more investigations are needed to explore this problem.

It is worth noting that our research results were not completely consistent with others. For example, Gu reported 1887 mRNAs and 960 lncRNAs were differentially expressed in PDLSCs after osteogenic induction for 7 days by RNA sequencing.^53^ As for the mRNA and lncRNA expression profiles in osteo‐differentiated hGMSCs, few studies were reported. The differences between the present study and others may be related to detection technology, individual differences of cells and so on. Anyway, the present results provide potential directions for molecular mechanism study. Further functional verification experiments are needed to confirm the function and mechanism of these mRNAs and lncRNAs, which is the goal of our future research.

## 5 CONCLUSIONS

This study analysed the common and specific differentially expressed mRNAs and lncRNAs in hPDLSCs and hGMSCs during osteogenic differentiation through microarray and bioinformatic methods, which provide clues to reveal the mRNAs‐lncRNAs‐based regulation for different osteo‐differentiation potentials of hPDLSCs and hGMSCs. Higher DKK1 expression in hGMSCs than in hPDLSCs after osteogenic induction is probably one reason for weaker osteo‐differentiation ability of hGMSCs, and inhibiting DKK1 is a potential method to improve the osteogenic differentiation ability of hGMSCs.

## CONFLICT OF INTEREST

The authors confirm that there are no conflicts of interest.

## AUTHOR CONTRIBUTIONS

**Linglu Jia:** Data curation (equal); Validation (equal); Writing‐original draft (equal). **Yunpeng Zhang:** Data curation (equal); Writing‐review & editing (equal). **Dongfang Li:** Validation (equal); Writing‐review & editing (equal). **Wenjing Zhang:** Validation (equal); Writing‐review & editing (equal). **Dongjiao Zhang:** Conceptualization (equal); Writing‐review & editing (equal). **Xin Xu:** Conceptualization (equal); Methodology (equal); Writing‐review & editing (equal).

6

## Supporting information

Table S1

Table S2

Table S3

Table S4

Table S5

Table S6

Table S7

Table S8

Table S9

Table S10

Table S11

Table S12

Table S13

## Data Availability

The data that supports the findings of this study are available in the supplementary material of this article.
